# On the Patronage of Quacks and Impostors by the Upper Classes of Society

**Published:** 1846-04

**Authors:** 


### IV. ON THE PATRONAGE OF QUACKS AND IMPOSTORS BY THE UPPER CLASSES OF SOCIETY.

BY THE EDITOR.

---

The following communication appeared in the 'Athenæum' of Feb. 28, 1846. It is here reprinted in the hope that, through the medium of the members of the profession, it may not only reach an additional number of that class of persons to whom it was, in the first instance, specially addressed, but may reach them with the further advantage of new illustrations and analogous commentaries, which it must be in the power of every medical man to supply. The few following prefatory remarks seem to afford not only a further reason for republishing it, but also for giving it a place among the communications to which this department of the Journal is at present devoted.

The evil which the subjoined narrative is intended to expose seems so far from decreasing with the progress of knowledge, that it would appear almost to keep pace with it. And it is worth the serious consideration of the members of the profession, whether this belief in the most palpable of absurdities may not, in some degree at least, be fostered by part of their own conduct.

When we consider, for instance, the knowledge which the public have of the kind of evidence of the powers of remedies which medical men are often satisfied with—the mystery which is still, not unfrequently, sometimes purposely, thrown around the real or supposed mode of action of medicines—the belief engendered by much of the ordinary practice, that nature is helpless in the cure of diseases, and the active interference of art necessary in all cases—and, lastly, the overweening confidence so constantly displayed in the potency of many medicaments of obscure action or of no action at all—there would certainly appear to be some reason for having a like fear with Macbeth, "that we but teach instructions which, being taught, return to plague the inventor." If men ignorant of every principle of science, especially medical science, are led, on what they must consider good authority, to regard the relation of sequence of events as the accurate exponent and characteristic of medicinal cure, there exists no good reason for surprise if they fall into the trap laid for them by the impostor, when they can urge in excuse that the grounds on which they proceed are as sound and secure in the one case as the other. We know that this is not so; because we know that our own knowledge, whatever be its amount, is real, and our good faith unquestionable, while the utter ignorance and roguery of the professed charlatans are as real and unquestionable; but the public can only be made to know this by having their minds enlightened as to the true principles on which therapeutics is based, and as to the actual amount of our positive knowledge and positive power, and being thus made to see and comprehend the true and unmistakeable distinctions between rational practice and empiricism, and between the honorable professors of legitimate medicine and the vile race of quacks with which the world in general, and this country in particular, is infested.

### MADEMOISELLE JULIE.

Omne animi vitium tanto conspectius in se
Crimen habet quanto major qui peccat habetur. (Juv. Sat. viii.)

Every now and then we read in the public prints of some wretched old woman brought before the police magistrate for practising, or pretending to practise, witchcraft, and therethrough swindling juvenile widows and love-sick maid-servants out of their shillings and sixpences.* Occasionally, also, we find parties of the same class and craft invading the province of the doctor, and doing "a snug little business" in the way of prescribing for, and of course curing, the diseases of all and sundry who may become their clients. The medium through which these wise women of the alleys and suburbs of this great city profess to become mistresses of the maladies of the unseen, is commonly a bit of rag from the clothing, a nail-paring, a lock of hair, or anything else connected with the person of the patient. The half-crown being paid, the nature of the malady is declared, and the means of cure specified. This is very various, according to the experience, the genius, or the fancy of the prescriber. Sometimes the disease is combated by what the learned would call *dynamic* means, such as words† or gestures, or the doing certain things at certain hours, or the handling of black or white cats, the plucking feathers from the tails of cocks, &c. At other times, the vulgar materials wherewith doctors work are put in requisition; especially those more obsolete sorts of drugs which, owing to the prime virtues of powerlessness and harmlessness, have come down to our times with undiminished fame from the days of the Asclepiades or before. Cures marvellous and manifold are thus wrought; cures, the result of which is never questioned; and which, to the philosophers of the alleys and attics, seem, and are, unquestionable. And no marvel. Have not these reasoners the very same grounds for their belief which satisfy their betters? The disease was declared, the remedy prescribed and administered, and the patient after a time got well. What can be more convincing? If, being ignorant of physic, they are ignorant of the fact that nature has the happy power of curing some diseases of her own mere motion; and if, having studied neither Bacon nor Locke, they confound sequence with consequence, the *post hoc* with the *propter hoc*,—can we blame severely, or at all, their loose logic or their halting reasoning? Should we not rather pity, and excuse, and forgive them, laying blame, if blame there be, on the lowliness of their lot and all its attendant circumscriptions, which make ignorance unavoidable, science impossible? Alas,

—— Knowledge to *their* eyes her ample page,
Rich with the spoils of time, did ne'er unroll.

Nor, looking to the influences of the same condition, the same circumstances, the same opportunities, the same causes, should we regard with too deep a disgust, or visit with too fierce an indignation, the poor wretches who thus practise on the ignorance and credulity of their humble neighbours. In one sense, knowledge may be said to be goodness as well as power; if it strengthens the intellectual faculties into wisdom, it strengthens the moral faculties into virtue. It has this tendency at least; and if it does not always do so, it often does so. Ought we, then, to feel surprise that among the children of penury and ignorance there are deceivers as well as dupes?

* The fact of the present paper having been written for a non-medical Journal will account for some peculiarities of manner and style, which will not fail to strike the professional reader.

† Sunt verba et voces, quibus hunc lenire dolorem
Possis, et magnam morbi deponere partem. (Hor. Ep. I, 1.)

But what shall we say for those who, without having any of the same grounds for excuse, exhibit the same intellectual debility, the same debasing credulity, the same lamentable ignorance and error? Could it be credited, if it were not known as a positive and melancholy truth, that it is by the upper classes of society, by our aristocracy, that quacks, charlatans, pretenders, and impostors of all sorts, are most especially patronized? Proofs of this fact, and the most pertinent illustrations, present themselves on every side. Indeed the thing is undeniable—is notorious. What is its explanation? Can it be aught else than this—that among a portion of this class of the community, with all their refined and fashionable culture and accomplishments, science and logic, scientific truths, and the modes of investigating them and judging of their nature, their evidence and value—are as little known as among their social antipodes? If such is the fact, it is one as melancholy to contemplate as it is deeply to be deplored: it is more—it is discreditable, unjustifiable, fraught with much present evil, and ominous of more.

I give the following brief narrative, as explaining and illustrating, and (I hope) justifying the observations and animadversions which precede. I leave to the reader all comment on the case. To me it seems to speak for itself, "with most miraculous organ," disclosing secrets of the most humiliating and portentous kind, in quarters where, least of all, such disclosures should be possible.

During the last six months there has been allocated in the near neighbourhood of the most fashionable precinct of the West-end, a certain young Frenchwoman, known by the name of Mademoiselle Julie, who has obtained a great reputation among our aristocracy as a curer of diseases. She is about twenty years of age, obviously from her manners and conversation of the lower orders of society, ill-educated, and indeed illiterate. She is accompanied by her mother, a person in manners and bearing even inferior to her daughter, and by a gentleman who is said to be the brother-in-law of the mother. These people at present occupy good furnished lodgings in a street opening into one of the West-end squares. Their principal operations are performed at home; but Mademoiselle also condescends to visit patients at their own houses, more especially those of high rank and title.

The system adopted by Mademoiselle Julie is too ingenious and too well calculated to attract attention from the class by whom she is patronized, to allow us to doubt that it has been adopted after mature consideration and with malice aforethought.

It is well known that the two most striking and attractive delusions of recent times, HOMŒOPATHY and MESMERISM, have met with especial favour and patronage from the upper classes of society in this country, and have, through their means chiefly, become in consequence fashionable and famous. The system of the fair Julie has the singular merit not only of combining these two celebrities, but of selecting their most attractive and agreeable parts, and separating them from all that is offensive and troublesome. Thus armed, thus accomplished, is it surprising that her success has been great, or that, from the first day of her descent upon the realms of fashion, she has gone on conquering and to conquer?

This is the system of our Wise-Woman of the West-end: The sick person cuts off a lock of her (or his) own hair "close to the head," places it, unprofaned by other touch, upon a piece of white silk, folds this with his (or her) own hand, and finally deposits it in an envelope of clean paper. This facile and self-executed rape of the lock is all that is required of the patient in the first instance. No doctor intrudes with his troublesome and disagreeable questions—no pulse need be felt—no tongue need be shown—no horrid *percussor* or more horrid *stethoscope* need frighten the gentle breast from its propriety. The lock is shorn, the deed is done; the dropped *Morning Post* is picked up, the new novel is resumed; the ripple of a moment vanishes, and the surface of life is tranquil as before. The next step is to convey the precious lock to the cell of the Wise-Woman, where the real business begins. This is transacted as follows: The uncle or mother of Julie magnetizes or mesmerizes her by some of the ordinary manipulations, and she falls asleep almost instantly (time is precious to those who are paid by the half-hour). The hair is then placed in her hand by the person who brings it; this person is put *en rapport* with her, by simply touching her hand once; she removes the covering from the mystical lock, takes it into her hand, and then commences a very active and elaborate process of rubbing, and squeezing, and picking it with the right hand, while it is held by the left; occasionally, also, she smells it. When this process has continued a few minutes, she begins to touch and press her own body with the fingers of the right hand, moving them from one place to another, sometimes rapidly, sometimes slowly, but finally dwelling preferably on one place, which she continues to press and manipulate more mystically and earnestly, and at last exclusively. It is then easily guessed that *here* is the site of the patient's principal malady, and the guess is soon verified by the words of the Pythoness. These words are waited for by the uncle, pen in hand, and are immediately committed to paper as they are uttered slowly, interruptedly, and in a subdued, sleepy tone. The record is made in the first person singular, as if the fair Julie were the patient. "I feel a pain,"—"I feel a sensation," &c., a mode of expression which is accounted for by the transcendent fact, of which both Julie and her *confrère* assure us, that through the mystic influence of the lock of hair by the intermingling of *its* (i. e. the patient's) *magnetic fluid* with her own, she, poor soul! is for the nonce made the recipient of all the aches, pains, sensations,—in short, of all the morbid symptoms of the unseen sufferer, who may, for anything she knows or cares, be hundreds of miles distant.

Good heaven, what a life of martyrdom must be that of poor Julie! To have one's poor carcase made the stage on which all the horrors that escaped from Pandora's box are to play their part—one after another, from morning to night; and, worse than all, a new one every hour! The very imagination of the thing is intolerable; what must be the reality? The conception of such an intrinsic monopoly by one poor body of all the ills that flesh is heir to, puts that of Dante to shame. The worst torments of the *Inferno* must yield to the Promethean sufferings of the unhappy Julie. And then, what inconceivable devotion to the cause of humanity, what unexampled fortitude, what heroic courage to dare and do all this, voluntarily, willingly, readily, cheerfully, yea eagerly! It is, of course, impossible to believe that into a mind capable of doing and suffering such things, the thought of fee or reward as compensation could enter; and, doubtless, the half-sovereign per *séance* and per lock, is accepted either in simple accordance with the practice of vulgar doctors, or for the purpose of being expended in relieving the sufferings of others, which assuredly none can know so truly and feel so surely as our poor Pythoness. But to return.

Having exposed the ills of one region, she passes to another, then to a third (as the case may be), and so on until she has given the full, true, and particular account of all the patient's diseased organs and their various symptoms. This is what the doctors call the *diagnosis* of the disease (viz. the settling its nature and name), which is followed by its *prognosis*, or exposition of its result; and, last of all, comes the *treatment*. This is set about as follows: A small box or tray containing upwards of two hundred tiny bottles is set before her. These bottles are those used by the Homœopathists, each containing its multitude of globules of medicated sugar of milk, with the name of the contained remedy pasted on each. She passes her fingers rapidly over the corks of this multitude of bottles, and selects three or four, when the rest are put aside. She sniffs at the selected few, and at length fixes on one: this is the certain remedy for the disease, if it is remediable, or its emollient, if it is incurable. The half-sovereign is then paid, and the *séance* breaks up.

During the whole course of the proceedings, Julie remains with her eyes nearly or wholly closed, and speaks in a subdued tone; but exhibits no special indication to the observer of being in any peculiar condition but what might be expected from any person performing the part that is performed by her. She converses freely with the person originally placed *en rapport* with her, and answers any question he may put in relation to the patient or to herself.

"My personal knowledge of Julie and her proceedings is limited to two visits on two successive days, recently paid to her at her lodgings in ——— street. These visits were paid at the suggestion of a gentleman of rank, for one of whose relatives Julie had prescribed; and who, although a believer in her marvellous doings, was yet anxious that one who had had somewhat more experience with the mesmerists should observe her proceedings and test her powers. I willingly consented to accompany this gentleman to the cell of the Wise-Woman, not, of course, to settle any doubts I myself entertained of the true character of the whole affair—for of this I had no doubts—but in hopes that something might occur that would disabuse one honorable mind, at least, if it did even help to break the degrading and despicable spell which had snared and bound the judgment of hundreds of his own high class, reducing them, in this respect, to the level of the lowest. I was aware of the risk I was running of helping to confirm, instead of exposing, their absurd infatuation—which would be the consequence of Julie's guesses happening to be right in the particular cases I was to submit to her. On the other hand, I thought that a few very simple precautions in the selection of the cases, and in the mode of presenting them, would turn the chances on my side. I need hardly say that I knew the pretended knowledge to be an impossibility; but I knew, at the same time, that the symptoms of diseases are so various and vague, and many of such uniform occurrence in disease, that it would not be very difficult, by an enumeration of more or fewer of these common or universal symptoms, to give a colour of accuracy where nothing of the kind existed. And in the cases which had been already reported to me as successful instances of Julie's powers, I perceived that this was the usual course of her proceeding. I selected my cases accordingly—cases strongly marked, thoroughly definite, and with such bold and characteristic features that the failure to state *these* must be admitted as a total failure, however much mention might be made of many other symptoms of an inferior or immaterial kind. And in order to satisfy my friends that no special pleading would be possible either on my part or theirs, I placed a memorandum of the nature of each case in a sealed envelope, to be opened at the close of the sitting, and compared with the written revelations of the fair seer. In doing this, I confess that I felt my position somewhat humiliating, as if I were still open to the suspicion of entertaining some doubts as to the real state of things. However, for the reasons given above I went on.

"I had prepared three cases of disease; but I only *consulted* the fair Julie for two—one on each day. I regret that I cannot give here the full and exact particulars of each case, as they are now lying before me in my own memoranda, and those taken down from the dictation of Julie;* but these are

* These are appended to the present reprint.

only suited to the pages of a medical journal. The following general outline, however, will suffice for my present purpose:

"*Case First*—was that of a girl of twelve years of age, who has a most horrible and disfiguring disease of *the mouth*, but is in *the most perfect health* in other respects. So said my sealed memoranda. Julie's *diagnosis*, now before me, is—that there is disease of the heart and lungs, and stomach and kidneys, with general debility, fever, &c. &c., but not one word respecting the actual disease! Upon being questioned as to the sex of the patient, she said the individual was a woman ('*une femme*,' not *fille*).

"*Case Second*—was that of *a man*, with an incurable disease of a peculiar kind, having its seat in the *left lung*, and who laboured under no other disease, except debility and general derangement of functions necessarily dependent on so severe a malady. Mademoiselle Julie's memorandum says not one word of any disease of the lungs or other organs of the chest, but places all the mischief at the other extremity of the body, and allocates the main disease in an organ not possessed by that half of the species to which the individual belongs! The hair was declared to be *a woman's*, and the disease one peculiar to the sex!

This, I think, is what in vulgar language is called a "clincher," and with it I take my leave of the subject of Mademoiselle Julie. If, after receiving this taste of the quality of their oracle, her fashionable patrons and patronesses still continue to frequent her shrine, accept her inspirations, and obey her behests, it is to be at least hoped that the police magistrate will henceforth visit with pity and forgiveness, and not with reproach and punishment, the vulgar witches of the suburban alleys, or their poverty-stricken and unlettered victims.

After what is above truly reported of the sayings and doings of Mademoiselle Julie, the reader is left to form his own judgment as to her precise bodily and mental condition while making her revelations. I will only say, that not an iota of evidence exists in favour of the alleged fact of her being in that peculiar state termed by adepts, mesmeric or magnetic sleep, or somnambulism. To mine and to all common eyes, she seemed simply to be a very zealous but bungling fortune-teller, as wide awake as her nature permitted, but with her eyes shut. I think the very mesmerists will hardly contend for the opposite view of the case; although the extent of her blunders ought by no means to deprive her of their good word and patronage.

Two more observations I will make before concluding, which, considered in all their bearings, tend, I think, to account for a good deal of Julie's success with the class of persons who consult her.

None but those who have given a good deal of attention to the subject, and seen much of proceedings of the sort now under notice, can believe the utter incapacity of the majority even of educated persons to appreciate evidence as to matters of fact. And when the parties engaged in the observation of the phenomena are unreasoning partisans of the doctrine involved in them (as they generally are), it is literally true that trifles light as air are to such persons confirmation strong as proofs of Holy Writ. Although it is almost profanation to quote Bacon on an occasion like this, still, as his great words tell strongly on the matter in hand, I venture to give them in corroboration of the remark just made:—"The light of the understanding," he says, "is not a dry or pure light, but drenched in the will and affections, and the intellect forms its knowledge accordingly; for what men desire should be true, they are most inclined to believe. . . . . . . When the mind is once pleased with certain things, it draws all others to consent and go along with them; and though the power and number of instances that make for the contrary are greater, yet it either attends not to them or despises them, or else removes and rejects them by a distinction, with a strong and pernicious prejudice to maintain the authority of its first choice unviolated." (Nov. Org.)

The other observation is this:—Had I given Julie, on my *first* visit, the lock of hair which I gave her on my *second*, her description of the disease (though really false) would assuredly have been regarded by her votaries as an additional proof of her omniscience: inasmuch as on *that* occasion she really did hit on the organ which was affected *in the other case!* On such slight chances do the fame and fortunes of the great oracles of the world depend. The difference of *sex* would have been regarded as of no importance, being readily "rejected and removed by a distinction."

---

### NOTES OF THE CASES.

### Case I.—*Dr. Forbes's Memorandum.*

C. H., a girl æt. 12, who, from previous fever and affection of the mouth from mercury (some years since I think), suffered sloughing of the lips and cheeks, caries of the jaw-bone, loss of teeth, &c., leaving behind the present affection—viz., inability to open the mouth wide, total inability to close the mouth at the angles of the lips, consequent continuous flow of saliva from the mouth, imperfect utterance, &c. The little girl is in the hospital (St. George's) awaiting an operation for the cure of this defect. She is in perfect good health otherwise.

### Case I.—*Mademoiselle Julie's Statement.*

C H. 21 Fevrier, 1846. Pulsations très vives au cœur. Les valvules du cœur sont pleines; c'est ce qui est la cause des pulsations. Il y a un douleur au coté gauche du poumon. L'épine dorsale est faible, douloureuse et fatiguée, principalment vers les reins. Les membranes de l'estomac sont très rouges: il y a comme un poids sur l'estomac: il y a aussi de l'irritation à l'estomac. Un peu de fièvre, causée par la faiblesse. Je sens le corps bien faible. Il y a des taches rouges au poumon gauche. Le dessous de la clavicule est douloureux. Je sens que le siège principale de la maladie est aux poumons et au cœur. Il n'y a aucune lésion au poumon. Je sens au milieu du dos autre chose que ce que j'ai dit; je ne puis pas bien le définir. Le sang au cœur n'est pas bon. Tous les organes de la poitrine sont malades, mais non pas d'une manière dangereuse. Le mauvais état du sang me fait voir la maladie comme étant grave. Plus je touche les cheveux, plus il me semble reconnaitre ceux d'une personne que j'ai déjà touchée. Je sens que les cheveux sont ceux d'une femme. La maladie est grave; mais non pas incurable.

### Case II.—*Dr. Forbes's Memorandum.*

W. G., a man aged 52. Disease of rather more than three years and a half standing. During all this time he has had a severe cough, and pain on the left side of the breast, between the nipple and collar bone, and towards the middle of the breast. Has had repeated hemoptysis (six times), generally slight, but sometimes severe. Never any fever. Is now greatly emaciated.

Has also lately a pain in the throat about the larynx. His disease is rather peculiar, and is supposed to be encephaloid tumour of the lung (the left), which must prove fatal in no long time.

CASE II.—*Mademoiselle Julie's Statement.*

W. G. 22 Fevrier, 1846. Irritation aux intestins; pésanteur dans le bas ventre. Je vois des taches rouges à l'intérieur du bas ventre. Cette partie a beaucoup d'inflammation: la vessie est très enflammée, et la peau de la partie supérieure est très épaissée. L'eau qui se forme aux reins est tres épaisse. Le foie est gonflé, et cause une sensation pénible. Il y a faiblesse dans les jambes; la personne ne peut pas marcher. Je sens des douleurs derrière la tête. La circulation du sang est mauvaise dans les intestins et dans les jambes; le sang est faible et pauvre dans tout le corps. La maladie principale est dans le bas ventre. Je sens que c'est une femme. Le sang est si décomposé que la guérison me serait peu probable. Il y a cancer à la matrice.

